# Eye Tracking as a Tool for Detecting Alzheimer's Disease in People With Down Syndrome

**DOI:** 10.1111/jir.13214

**Published:** 2025-02-10

**Authors:** Brianna Piro‐Gambetti, Sharon Krinsky‐McHale, Cynthia Kovacs, Benjamin Handen, Bradley Christian, Charles M. Laymon, Davneet Minhas, Weiquan Luo, Dasoo Milton Yoon, Victoria L. Fleming, Emily Schworer, Heather Kirkorian, Sigan L. Hartley, Alzheimer Biomarkers Consortium‐Down Syndrome

**Affiliations:** ^1^ Waisman Center University of Wisconsin‐Madison Madison Wisconsin USA; ^2^ Department of Psychology New York State Institute for Basic Research in Developmental Disabilities New York New York USA; ^3^ Department of Psychiatry University of Pittsburgh Pittsburgh Pennsylvania USA; ^4^ Department of Radiology University of Pittsburgh Pittsburgh Pennsylvania USA; ^5^ Department of Human Development and Family Studies University of Wisconsin‐Madison Madison Wisconsin USA

**Keywords:** Alzheimer's disease, biomarkers, cognition, Down syndrome, eye tracking, memory

## Abstract

**Background:**

Adults with Down syndrome (DS) experience an increased risk of Alzheimer's disease (ad). Valid cognitive assessments for adults with DS with severe/profound intellectual disability (ID) are needed. It is unclear whether eye tracking is feasible for detecting ad in DS.

**Method:**

Fifty‐three adults with DS completed a visual paired comparison (VPC) task, a battery of cognitive measures, and underwent PET scanning. Study partners reported on the participant's dementia symptoms. Bivariate correlations assessed associations between eye‐tracking metrics and ad‐related pathology and symptomatology. Analyses included the full sample (*n* = 53) and a subgroup with an IQ ≤ 45 (*n* = 33).

**Results:**

Greater fixation duration during the habituation phase was associated with better cognitive performance on the Modified Cued Recall Test (mCRT) (intrusions: *r* = −0.39, *p* = 0.011) and less PET tau (*r* = −0.47, *p* = 0.014). Larger saccadic amplitudes during the test phase were associated with younger age (*r* = −0.45, *p* < 0.001), better cognitive performance on the mCRT (total: *r* = 0.31, *p* = 0.041; intrusions: *r* = −0.33, *p* = 0.032) and less PET Aβ (*r* = −0.40, *p* = 0.025). Greater preference to fixate on the novel image was associated with fewer dementia symptoms (count: *r* = −0.44, *p* = 0.002; duration: *r* = −0.38, *p* = 0.009). This pattern of significance remained for the subgroup with lower IQ scores.

**Conclusion:**

The VPC task is a potentially useful method for assessing ad‐related cognitive impairments in adults with DS across varying ID levels.

## 1 Introduction

Valid measures for tracking early cognitive decline related to Alzheimer's disease (ad) in people with Down syndrome (DS) are critically needed. Such measures are essential for guiding screening decisions and evaluating treatment efficacy in ad therapeutic trials. The prevalence of ad in DS is ~90% by age 70 (McCarron et al. 2017), due to the triplication of the amyloid precursor protein gene located on chromosome 21, resulting in the overexpression of amyloid‐β (Aβ) (Mann and Esiri 1989; Wiseman et al. 2015). Deposition of Aβ plaques is observed via positron emission tomography (PET) in individuals with DS in their 30s (Lao et al. 2016; Keator et al. 2020) and followed by neurofibrillary tangles in their 40s and 50s (Zammit et al. 2024), with the average age of dementia in the early to mid‐50s (Iulita et al. 2022). Direct measures of cognitive functioning have been identified as valid or promising for tracking early ad‐related cognitive decline in adults with DS (Krinsky‐McHale et al. 2022; Videla et al. 2022). Many of these measures, however, have poor testing properties in adults with DS with IQs below 40 or even 45 (Benejam et al. 2020; Esbensen et al. 2017; Krinsky‐McHale et al. 2020; Videla et al. 2022).

Across commonly used measures of ad‐related cognitive decline, problems with feasibility, floor effects, narrow range and poor sensitivity for capturing symptomatic ad clinical stages are reported for adults with DS with lower moderate to severe/profound intellectual disability (ID). For example, on the Rapid Assessment for Developmental Disabilities (RadD; Hom et al. 2021), floor effects increased and sensitivity to detecting early‐stage AD decreased when used with adults with DS with moderate to profound ID compared to mild ID (Walsh et al. 2007; Walsh et al. 2015). Similarly, the National Institutes of Health Toolbox Cognition Battery is not valid in individuals with a mental age ≤ 3–6 years (Hessl et al. 2016). The Modified Cued Recall Test (mCRT; Devenny, Krinsky‐McHale, and Adetoki 2018), a measure of episodic memory, has robust associations with biomarkers of early ad pathology (Hartley et al. 2022, 2023) and is highly sensitive and specific to mild cognitive impairment (MCI) and ad dementia in adults with DS (Benejam et al. 2020; Krinsky‐McHale et al. 2022; Videla et al. 2022) but suffers from floor effects in individuals with severe/profound ID (Benejam et al. 2020). Similarly, the Wechsler Block Design subtest (Wechsler 2004), even with the Haxby downward extension (Haxby 1989), has floor effects in adults with DS with severe/profound ID (Krinsky‐McHale et al. 2020). Not having validated direct measures of cognition that are appropriate for the broad range of premorbid ID levels evident in DS will delay ad diagnosis in addition to serving as a barrier for accessing treatments and being included in ad therapeutic trials.

Eye tracking during information processing offers a noninvasive method for assessing cognitive performance independent of verbal ability and does not require an understanding of complex instructions (Tadokoro et al. 2021; Wilcockson et al. 2019). Within the general population, eye tracking has been used to assess ad‐related cognitive decline (Chau et al. 2015; Crawford and Higham 2016; Zola et al. 2013). Particularly, visual paired comparison (VPC) tasks are used to assess learning and visual memory through infrared eye tracking (Helo et al. 2014; Tobii 2023). The VPC task has long been used in primate (e.g., Fantz 1958; Pascalis et al. 2009) and infant (e.g., Beckner et al. 2023; Krinsky‐McHale 1995) research and has strong reliability and good construct validity with other measures of learning and memory. During a habituation phase, participants are shown two identical images (habituation images). This is followed by the test phase, in which the familiar image (previously seen habituation image) is shown along with a novel image. Visual memory is assessed by whether the participant exhibits a preference for the novel image as opposed to the familiar image; greater fixation duration or a higher number of fixations on the novel image indicates better learning and memory. Using a VPC task in a general population adult sample, Lagun et al. (2011) distinguished visual memory performance between age‐matched healthy adults from those with MCI with 87% accuracy, 97% sensitivity and 77% specificity. Zola et al. (2013) found that scores on a VPC task predicted which healthy adults progressed to MCI (vs. remained cognitively stable) and which adults with MCI progressed to dementia (vs. remained MCI) up to 3 years later.

In eye‐tracking paradigms, visual saccades, often measured in amplitude, are defined as rapid eye movements between fixations (Helo et al. 2014; Molitor, Ko, and Ally 2015; Tobii 2023) and are an established biomarker of cognitive ability. Larger saccades and shorter fixations signal ambient attention whereby an individual learns the overall gist of the scene (Helo et al. 2014; Tatler and Vincent 2008). In contrast, smaller saccades and longer fixations indicate focal encoding of the scene in a detailed and complex way (Pannasch et al. 2008; Tatler and Vincent 2008), a skill that improves throughout childhood into adolescence (Aring et al. 2007; Helo et al. 2014). In general population samples, older adults with dementia exhibit larger saccades and shorter fixations relative to healthy controls (Ionescu et al. 2023; Pavisic et al. 2017). Moreover, shorter fixations (Ionescu et al. 2023; Tadokoro et al. 2021) and larger saccades (Yang et al. 2013) were associated with worse performance on the Mini Mental State Examination (Folstein, Folstein, and McHugh 1975). These findings suggest that individuals with ad dementia use a less complex ambient mode of processing and struggle fixating on details.

Studies that examined eye‐tracking metrics in DS focused on infants and children (e.g., van Herwegen et al. 2019; Schworer et al. 2021) and suggest that eye movements are correlated with indices of learning and memory. No studies, to our knowledge, have examined whether eye‐tracking metrics are useful for tracking dementia in adults with DS. VPC tasks place minimal demand on the participant and involve a passive (vs. active) viewing task, thus providing a potentially less cumbersome approach than the traditional measures currently used to assess ad‐related impairments. The goal of the current study was to determine the validity of an eye‐tracking VPC task for assessing ad‐related cognitive impairments and biomarkers of ad pathology in 53 adults with DS (aged 26–57 years) who ranged from mild to severe/profound premorbid ID. Study aims were to (1) examine associations between VPC eye‐tracking metrics and ad‐related cognitive impairments via directly‐administered and informant‐reported measures and (2) evaluate the relation between VPC eye‐tracking metrics and PET biomarkers of Aβ and tau. Analyses were run with the full sample and then with only adults with DS with IQs ≤ 45. In line with general population, shorter duration of fixations and larger saccades during the habituation phase (initial learning) or for the novel (vs. familiar) images during the test phase were predicted to be associated with older age, higher PET Aβ and tau, presence of MCI or dementia and poorer cognitive functioning.

## 2 Method

### 2.1 Participants

Fifty‐three adults with DS were recruited from the Alzheimer Biomarker Consortium in DS (ABC‐DS; Handen et al. 2020) or an auxiliary study that recruited adults with DS with IQs ≤ 45. Inclusion criteria included (1) aged ≥ 25 years; (2) genetically confirmed trisomy 21; (3) vision (with or without correction) within 20/50 bilaterally and intact ocular motility; (4) study partner capable of reporting on clinical symptoms and adaptive functioning; and (5) no untreated physical or mental health conditions that could impact neuropsychological testing (e.g., recent stroke or untreated severe hypothyroidism). Adults with DS deemed to have dementia in ABC‐DS (see Handen et al. 2020) were not administered the full battery of cognitive measures by study design and thus were excluded from the present study.

The 53 participants (32 [60%] male and 21 [40%] female) had a mean chronological age of 38.1 years (SD = 7.8) and mental age equivalence of 5.0 years (SD = 1.4; range: 2.0–8.8). IQ scores ranged from 47 to 55 on the SB5 and 40 to 75 on the KBIT‐2 with a mean IQ of 48.2 (*SD* = 9.3). Thirty‐three (62%) participants had an IQ ≤ 45 or SB5 standard IQ of 47 with a mental age equivalent of ≤ 5.00 years. Table 1 provides sample sociodemographics for the full sample, those with an IQ ≤ 45 and those with an IQ > 45, separately. All (100%) participants completed the VPC task. PET Aβ and tau values were available for the 33 participants recruited from the ABC‐DS study.

**TABLE 1:** Sample sociodemographics.

	Full sample (*n* = 53)	IQ ≤ 45 (*n* = 33)	IQ > 45(*n* = 20)
Age in years, *M* (SD)	38.1 (7.8)	39.9 (8.4)	35.2 (5.6)
Mental age equivalence in years, *M* (SD)	5.0 (1.4)	4.2 (0.8)	6.3 (1.3)
Female, *n* (%)	21 (40%)	10 (30%)	11 (55%)
Wore eyeglasses, *n* (%)	32 (60%)	20 (61%)	12 (60%)
Additional visual impairment, *n* (%)	16 (30%)	8 (24%)	5 (25%)
Cataracts	10 (19%)	6 (18%)	2 (10%)
Astigmatism	2 (4%)	1 (3%)	1 (5%)
Lazy eye	1 (2%)	0 (0%)	0 (0%)
Strabismus	1 (2%)	0 (0%)	1 (5%)
Keratoconus	2 (4%)	1 (3%)	1 (5%)
Karyotype, *n* (%)			
Full trisomy	47 (89%)	31 (94%)	19 (95%)
Mosaicism	1 (2%)	0 (0%)	0 (0%)
Translocation	4 (8%)	3 (9%)	1 (5%)
N/A	1 (2%)		

*Note:* Mental age equivalency was calculated via either the Stanford Binet Intelligence Scales, Fifth Edition (Roid and Pomplun 2012), or the Kaufman Brief Intelligence Test Second Edition (K‐BIT2; Kaufman and Kaufman 2004).

### 2.2 Procedure

The study had IRB approval through the University of Wisconsin‐Madison. Participants with DS, or their legal guardian, provided informed written consent. If a legal guardian provided consent, the participant with DS provided written assent. Prior to obtaining consent/assent, the study was described in detail, and a talk‐back method was used to ensure understanding. Participants with DS completed a 2.5‐h neuropsychological battery to screen for early ad‐related cognitive decline (for details of the full neuropsychological battery, see Handen et al. 2020), underwent MRI and PET imaging and completed a 10‐min VPC task. Analyses for the present study focused on three neuropsychological measures from the larger battery due to their sensitivity to early onset ad in DS (Hartley et al. 2022; Krinsky‐McHale et al. 2022). The study partner reported on the participant's sociodemographics and dementia symptoms.

### 2.3 Measures

#### 2.3.1 Sociodemographics

Study partners reported on the participant's date of birth (converted to age) and biological sex (1 = male and 2 = female). Blood karyotype was used to determine trisomy 21 type (1 = full trisomy, 2 = mosaic and 3 = translocation). Standardised scores for overall IQ were obtained via direct administration of the Stanford‐Binet, Fifth Edition (SB5), Abbreviated Battery (Roid and Pomplun 2012) or the Kaufman Brief Intelligence Test Second Edition (K‐BIT2; Kaufman and Kaufman 2004). The lowest possible IQ score on the SB5 Abbreviated battery is < 47; thus, mental age of > 5.00 years was used to identify individuals with DS with IQs > 45 on the SB5.

#### 2.3.2 Visual Paired Comparison Task

Participants with DS were seated in a windowless room 55–65 cm way from the Tobii Spectrum Pro eye tracker (Version 2.0.10) monitor (60.45 cm, 1920 × 1080‐pixel screen [EIZO FlexScan EV2451]). Participants had 34 cm × 26 cm head movement freedom whereby gaze samples were detected by at least one eye. Blink recovery time occurred immediately; if the pupil was occluded for a few hundred milliseconds or less, tracking continued once the pupil was visible again. Gaze recovery time or the ability to detect the pupil and gaze after losing track of the participant's eyes was less than 150 ms. The session began with a 9‐point calibration. No participant reached a precision of < 1° on all 9 points. Due to the large dimensions of our Areas of Interest (AOIs; 15.24 cm × 15.24 cm boxes around images), the accuracy and precision data were deemed acceptable. Our task was not dependent on specific areas of fixation within novel versus habituation images. During calibration, 38% of participants had an average accuracy of < 10 mm, 53% had < 20 mm and 79% had < 45 mm. In terms of precision (spread of gaze points), 27% of participants had an average RMS score < 10 mm, 41% had < 20 mm and 85% had < 55 mm. There was not a significant correlation between accuracy or precision on the calibration task and cognitive measures (*p*s > 0.05). Thirty‐two (60%) participants wore eyeglasses, and 16 (30%) had additional visual impairments (e.g., cataracts, strabismus and keratoconus). The presence of eyeglasses was not significantly associated with gaze accuracy (*r* = −0.02, *p* > 0.05) or precision (*r* = 0.20, *p* > 0.05) nor was the presence of an additional visual impairment (accuracy: *r* = 0.02, *p* > 0.05; precision: *r* = −0.16, *p* > 0.05).

Eye movements were recorded using binocular bright‐ and dark‐pupil tracking technique, and data were analysed using Tobii Pro Lab software (Version 1.207.44884) at 300‐Hz sampling rate and a manufacturer‐reported gaze position accuracy of 0.3° of visual angle. Within the Tobii Pro Lab software, the Tobii I‐VT fixation filter calculated fixation and saccade data. Variables of interest included the number of fixations (fixation count), length of fixation (fixation duration) and saccadic amplitude (i.e., distance from fixations preceding and following the saccade) (Tobii 2023).

**FIGURE 1:**
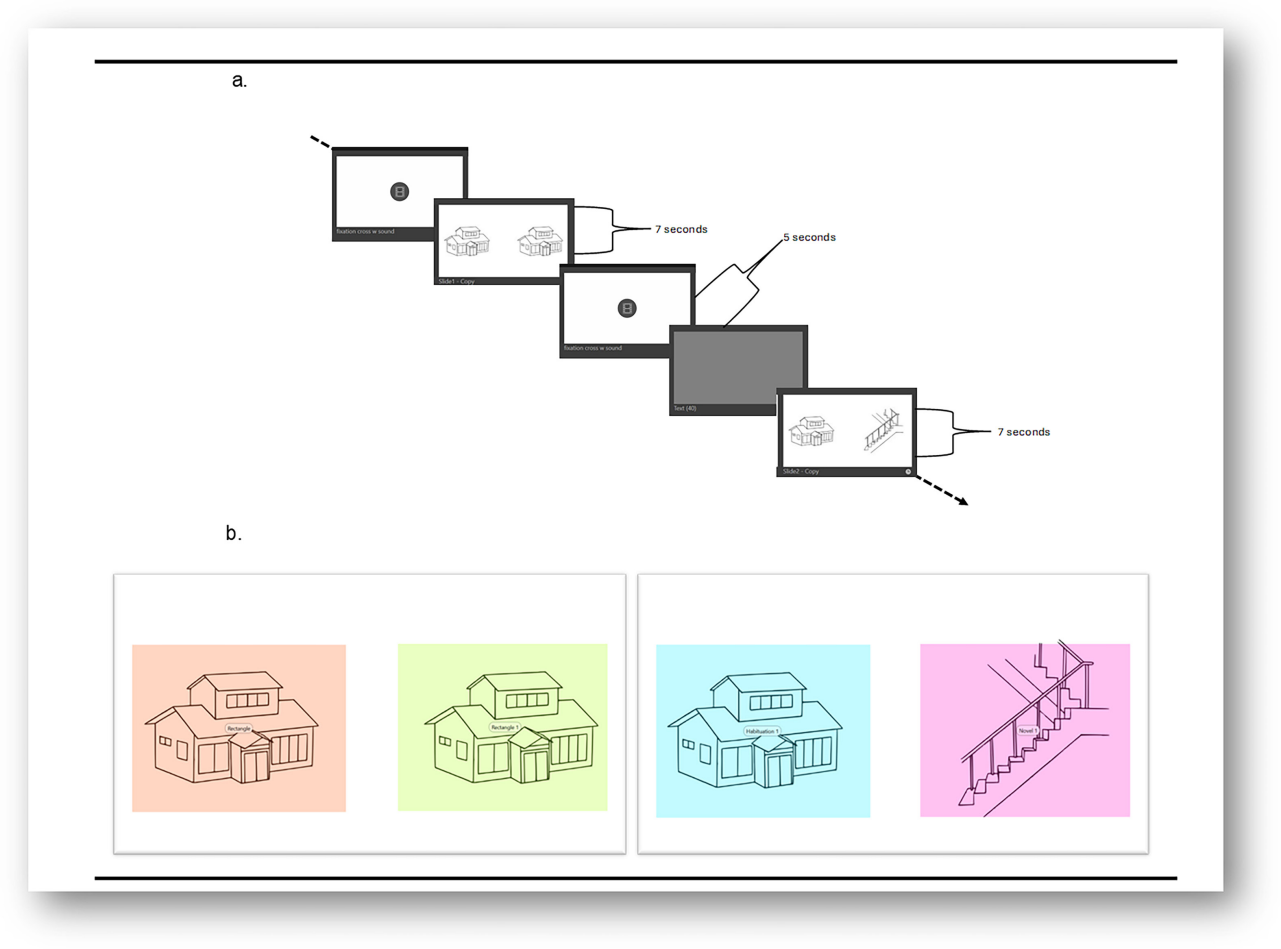
(a) Sample task sequence. (b) Areas of Interest (AOIs). AOIs include the entire coloured box.

Participants were instructed to place their hands in their lap and look at the screen. The VPC task had 20 trials, each with a habituation, delay and test phase. The habituation phase involved two identical images, side by side on the screen for 7 s. This was followed by a black screen. A chime sounded, and a fixation cross appeared in the middle of the screen (totalling 5 s) before the testing phase. The testing phase involved two different images—familiar image (in habituation phase) and novel image (not previously seen)—side by side on the screen for 7 s. Images were black and white and of comparable size, line thickness and detail. See Figure 1 for an example of AOIs. The placement of the familiar versus novel image (e.g., right vs. left) was counterbalanced across trials. Fixation duration was examined for the habituation phase to assess learning. The visual preference score (i.e., relative fixation count and duration for the novel versus habituation image) assessed memory.

#### 2.3.3 Cognitive Measures

Participants with DS completed cognitive measures shown to detect early ad‐related impairments in DS (Benejam et al. 2022; Startin et al. 2019). Episodic memory was assessed using the modified Cued Recall Test (mCRT; Zimmerli and Devenny 1995), which is sensitive to ad pathology (Hartley et al. 2022) and MCI and dementia in DS (Krinsky‐McHale et al. 2022). Participants first learn 12 items (e.g., star) and their respective categories (e.g., something in the sky). They are asked to freely recall items and then given a category cue. The mCRT total is the sum of free and cued items across three trials. The mCRT intrusion score is the number of incorrectly recalled items during cued trials.

Motor coordination and planning was assessed via the Purdue Pegboard (Vega 1969). Participants are given 30 s to place pegs into holes as quickly as possible first using their dominant hand and then nondominant hand. Purdue Pegboard scores are negatively associated with PET Aβ burden in adults with DS (Hartley et al. 2017).

Visuospatial ability was measured using the Wechsler Intelligence Scale for Children, Fourth Edition (WISC‐IV; Wechsler 2004), Block Design and Haxby downward extension (Haxby 1989). Participants are instructed to create designs using blocks. The WISC‐IV Block Design can distinguish between adults with DS with versus without dementia (e.g., Krinsky‐McHale et al. 2020).

#### 2.3.4 Dementia Symptoms

Dementia symptoms were assessed via study partner report using the Dementia Questionnaire for People with Learning Disabilities (DLD; Evenhuis 2018). The DLD measures ad‐related cognitive (Sum of Cognitive Score; DLD‐SCS) and social (Sum of Social; DLD‐SOS) declines and can differentiate adults with DS with versus without ad dementia (Koehl et al. 2020). This informant‐reported measure has good testing properties in adults with DS with severe/profound ID (Wallace et al. 2021).

#### 2.3.5 MRI and PET Scans

Participants with DS underwent MRI and PET imaging scans. Assessment of Aβ load was performed using the tracer [^11^C]PiB, and [18F]AV‐1451 was used for the assessment of tau. Each tracer was administered as 20‐ to 30‐s bolus IV injection followed by a saline flush. PET images were collected in 5‐min frames, inspected on a frame‐by‐frame basis and corrected for motion. Images were acquired 50‐ to 70‐min postinjection for [^11^C] PiB and 80–100 min for [^18^F]AV‐1451. Data were reconstructed using iterative methods corrected for deadtime, attenuation, scatter and radioactive decay.

#### 2.3.6 PET Image Processing

For processing of both the Aβ and PET data, each participant's T1 MRI was the anatomical reference. Thus, each PET scan was registered to their T1 image. Processing of Aβ followed the centiloid procedure described by Klunk et al. (2015) using SPM8 software (https://www.fil.ion.ucl.ac.uk/spm/). Briefly, T1 MRI scan was warped to match the 152‐subject template of the Montreal Neurological Institute (MNI152), and the warping parameters obtained were used to co‐warp the (registered) [^11^C]PiB image. The average concentration of [11C]PiB within the standard global region (Klunk et al. 2015) was determined using the global region of interest (ROI) mask, defined on MNI152. The ROI samples frontal, temporal and parietal cortices and precuneus, anterior striatum and insular cortex (available at https://gaain.org/). Global concentration was normalised by dividing by the whole cerebellum concentration, obtained using whole cerebellum ROI. Normalised concentrations were converted to centiloid values using the published linear + constant transformation specified for [11C]PiB (Klunk et al. 2015).

To produce values for the tau analysis, the [18F] AV‐1451 tau images were registered to the T1 MRI using PMOD software (https://www.pmod.com/). FreeSurfer (FS) 5.3 (http://surfer.nmr.mgh.harvard.edu/) was used to section each unwarped T1 image into the Desikan‐Killiany atlas regions (Desikan et al. 2006). The regions were applied to the registered PET. A volume weighted average of concentrations within a set FS‐based components, normalised to the cerebellar cortex, determined SUVR of [18F]AV‐1451 in the Mayo composite region (Jack et al. 2017).

### 2.4 Data Analysis Plan

Analyses were conducted in SPSS (Version 29.0.1.0). The distribution of eye‐tracking metrics was evaluated, and all variables displayed normal distribution for both the full sample and the subgroup with lower IQ scores (full sample skew range: 0.062 to 0.807; full sample kurtosis range: −0.549 to 0.988; subgroup skew range: 0.293 to 0.831; subgroup kurtosis range: −0.862 to 0.760). Eye‐tracking metrics of interest included: (1) fixation duration on habituation phases; (2) saccadic amplitude for novel image during the test phases; and (3) visual preference for the novel (vs. familiar) image during the test phases (fixation count and duration). Gaze samples (i.e., percentage of correctly identified eye‐tracking samples used to calculate gaze points) were examined, and follow‐up analyses were conducted on participants who had an average gaze sample ≥ 50%. In order to understand if eye tracking is sensitive to ad biomarker and cognitive change in adults with DS with both higher and lower IQ scores, we ran independent *t* tests to compare eye‐tracking metrics between participants with IQ ≤ 45 and participants with IQ > 45. Bivariate Pearson or point‐biserial correlations examined the association between sociodemographics (age, sex and mental age equivalence) and eye‐tracking metrics. Bivariate correlations also examined the association between eye‐tracking metrics and measures of ad symptoms and pathology (PET Aβ and tau). Correlations were then rerun including only participants with IQ ≤ 45. Gaze plots from the Tobii Pro Lab software were reviewed.

## 3 Results

Table 2 provides means and SD for main variables for the full sample. During the test phase, on average, participants had a significant but slight preference for the novel image (vs. familiar image) (*t*[52] = −1.76, *p* = 0.042). There was not a significant difference in fixation *duration* for the novel image (vs. familiar image) (*t*[52] = −0.85, *p* = 0.201). The mean number of fixations on the familiar images was 5.9 (*SD* = 2.4) and 6.5 (*SD* = 2.7) on the novel images. Mean duration of fixation on the familiar images was 1.9 s (*SD* = 0.8) and 2.0 s (*SD* = 0.8) on the novel images. Mean number of saccades on the familiar images was 2.1 (*SD* = 1.7) and 2.5 (*SD* = 2.3) on the novel images. Mean saccadic amplitude for the familiar images was 4.5° (SD = 1.6) and 4.6° (SD = 1.6) for the novel images, with maximum amplitudes > 8° (familiar images: 8.5°; novel images: 9.0°).

**TABLE 2:** Means and standard deviations of study variables.

	Full sample	Lower IQ group^a^	Higher IQ group^b^
	*M* (*SD*)	*M* (*SD*)	*M* (*SD*)
Saccadic Amplitude_Familiar Image, degrees	4.5 (1.6)	4.5 (1.6)	4.5 (1.7)
Saccadic Amplitude_Novel Image, degrees	4.6 (1.6)	4.6 (1.8)	4.6 (1.3)
Fixation Count_Familiar Image	5.9 (2.4)	5.7 (2.7)	6.3 (1.9)
Fixation Count_Novel Image	6.5 (2.7)	6.4 (2.9)	6.7 (2.6)
Fixation Duration_Familiar Image, seconds	1.9 (0.8)	1.8 (0.9)	2.1 (0.6)
Fixation Duration_ Novel Image, seconds	2.0 (0.8)	2.0 (0.9)	2.1 (0.7)
DLD‐SOS^c^	5.9 (5.7)	7.1 (6.6)	3.9 (2.9)
DLD‐SCS^d^	5.5 (6.9)	8.4 (7.4)	0.9 (1.2)
mCRT Total^e^	29.7 (10.1)	26.0 (11.2)	35.5 (3.2)
mCRT Intrusions^f^	3.6 (6.1)	5.4 (7.2)	0.8 (1.2)
Purdue Pegboard Dominant Hand^g^, (number of pegs correctly placed)	6.2 (2.3)	5.4 (2.0)	7.5 (2.4)
Purdue Pegboard Nondominant Hand^h^, (number of pegs correctly placed)	6.6 (2.6)	5.8 (2.5)	7.9 (2.3)
Block Design^i^	23.0 (11.9)	17.6 (10.9)	31.7 (7.6)
PET Aβ^j^, centiloids	14.0 (19.6)	19.5 (22.4)	4.5 (7.5)
PET tau	1.2 (0.1)	1.2 (0.1)	1.1 (0.09)

^a^ IQ ≤ 45.

^b^ IQ > 45.

^c^ Dementia Questionnaire for People with Learning Disabilities sum of social score (Evenhuis 2018).

^d^ Dementia Questionnaire for People with Learning Disabilities sum of cognitive score (Evenhuis 2018).

^e^ Modified Cued Recall Test total score (Zimmerli and Devenny 1995).

^f^ Modified Cued Recall Test cued intrusions score (Zimmerli and Devenny 1995).

^g^ Purdue Pegboard dominant hand score (Vega 1969).

^h^ Purdue Pegboard nondominant hand score (Vega 1969).

^i^ Wechsler Intelligence Scale for Children Block Design subtest (Wechsler 2004) with Haxby downward extension (Haxby 1989).

^j^ PET amyloid‐beta.

### 3.1 Correlates of Eye‐Tracking Data

Bivariate correlation results for associations between eye‐tracking metrics and sociodemographics, ad pathology and ad symptomatology are in Table 3. Confidence intervals for the bivariate correlations are in Table 4. Lower chronological age was significantly associated with greater average saccadic amplitude for the novel image (*r* = −0.45, *p <* 0.001). Neither mental age equivalence nor biological sex was significantly associated with eye‐tracking metrics (*p* > 0.05). To address Study Aim 1, the associations between VPC metrics and ad‐related cognitive impairments were examined. During testing phases, greater average saccadic amplitude for the novel image was significantly associated with mCRT Intrusions (*r* = −0.33, *p* = 0.032) and mCRT Total (*r* = 0.31, *p* = 0.041). During habituation phases, the average fixation duration was significantly correlated with mCRT Intrusions (*r* = −0.39, *p* = 0.011). There was a significant association between both visual preference variables (fixation count: *r* = −0.44, *p* = 0.002; fixation duration: *r* = −0.38, *p* = 0.009) and the DLD‐SOS. The Block Design total was not significantly correlated with eye‐movement metrics. To address Study Aim 2, the relationship between VPC metrics and PET biomarkers of Aβ and tau was examined. Saccadic amplitude for the novel images (*r* = −0.40, *p* = 0.025) was associated with PET Aβ. Fixation duration on the habituation phases was associated with PET tau (*r* = −0.47, *p* = 0.014).

**TABLE 3:** Pearson correlations.

	Full sample				Lower IQ group^a^			
	*N* = 53				*N* = 33			
	Fixation duration (habituation phase)	Saccadic amplitude_novel (test)	Prefernce^b^_fixation count (test)	Preference_fixation duration (test)	Fixation duration (habituation phase)	Saccadic amplitude_novel (test)	Preference_fixation count (test)	Preference_fixation duration (test)
Age	−0.25	−0.45***	0.12	0.11	−0.25	−0.48**	0.18	0.21
Mental Age	0.17	−0.10	−0.18	−0.25	0.21	−0.11	−0.02	−0.08
Sex	0.12	0.04	−0.19	−0.17	0.03	0.16	−0.23	−0.21
DLD‐SOS^c^	−0.01	0.09	−0.44**	−0.38*	−0.00	0.13	−0.64***	−0.60***
DLD‐SCS^d^	−0.17	−0.01	−0.26	−0.19	−0.10	−0.01	−0.41*	−0.35
mCRT total^e^	0.12	0.31*	0.05	0.01	0.01	0.41*	0.10	0.08
mCRT intrusions^f^	−0.39**	−0.33*	−0.04	−0.01	−0.40*	−0.39*	−0.08	−0.06
P.Pegboard.Dom^g^	0.21	0.03	−0.00	−0.08	0.19	0.05	0.16	0.11
P. Pegboard.Non^h^	0.07	−0.20	0.26	0.17	0.06	−0.23	0.36	0.28
Block Design^i^	0.13	−0.05	−0.00	−0.12	0.17	0.07	0.14	0.07
Aβ^j^	−0.24	−0.40*	0.23	0.29	−0.35	−0.55*	0.18	0.26
Tau^k^	−0.47**	−0.31	0.001	0.05	−0.59*	−0.57*	−0.10	−0.03

^a^ IQ ≤ 45.

^b^ Preference scores indicate preference to fixate on the novel image of the test phase.

^c^ Dementia Questionnaire for People with Learning Disabilities sum of social score (Evenhuis 2018).

^d^ Dementia Questionnaire for People with Learning Disabilities sum of cognitive score (Evenhuis 2018).

^e^ Modified Cued Recall Test total score (Zimmerli and Devenny 1995).

^f^ Modified Cued Recall Test cued intrusions score (Zimmerli and Devenny 1995).

^g^ Purdue Pegboard dominant hand score (Vega 1969).

^h^ Purdue Pegboard nondominant hand score (Vega 1969).

^i^ Wechsler Intelligence Scale for Children Block Design subtest (Wechsler 2004) with Haxby downward extension (Haxby 1989).

^j^ PET amyloid‐beta.

^k^ PET tau.

* *p* < .05.

** *p *< .01.

*** *p* < .001.

**TABLE 4:** Confidence intervals.

	Full sample				Lower IQ group^a^			
	*N* = 53				*N* = 33			
	Fixation duration (habituation phase)	Saccadic amplitude_novel (test)	Prefernce^b^_fixation count (test)	Preference_fixation duration (test)	Fixation duration (habituation phase)	Saccadic amplitude_novel (test)	Preference_fixation count (test)	Preference_fixation duration (test)
Age	[−0.50, 0.03]	[−0.64, −0.20]	[−0.18, 0.39]	[−0.19, 0.38]	[−0.55, 0.12]	[−0.70, −0.15]	[−0.21, 0.52]	[−0.18, 0.54]
Mental age	[−0.11, 0.42]	[−0.36, 0.17]	[−0.44, 0.11]	[−0.50, 0.03]	[−0.11, 0.55]	[−0.44, 0.24]	[−0.39, 0.36]	[−0.44, 0.31]
Sex	[−0.16, 0.39]	[−0.22, 0.32]	[−0.45, 0.10]	[−0.43, 0.13]	[−0.33, 0.38]	[−0.18, 0.49]	[−0.56, 0.16]	[−0.54, 0.18]
DLD‐SOS^c^	[−0.29, 0.27]	[−0.20, 0.34]	[−0.65, −0.18]	[−0.60, −0.10]	[−0.36, 0.35]	[−0.24, 0.44]	[−0.82, −0.36]	[−0.80, −0.30]
DLD‐SCS^d^	[−0.43, 0.12]	[−0.31, 0.23]	[−0.51, 0.03]	[−0.45, 0.10]	[−0.44, 0.26]	[−0.38, 0.30]	[−0.68, −0.04]	[−0.64, 0.02]
mCRT total^e^	[−0.20, 0.41]	[0.02, 0.56]	[−0.27, 0.37]	[−0.31, 0.33]	[−0.39, 0.40]	[0.02, 0.68]	[−0.33, 0.50]	[−0.36, 0.48]
mCRT intrusions^f^	[−0.62, −0.10]	[−0.57, −0.04]	[−0.36, 0.28]	[−0.33, 0.31]	[−0.69, −0.01]	[−0.67, −0.01]	[−0.48, 0.36]	[−0.47, 0.38]
P.Pegboard.Dom^g^	[−0.08, 0.46]	[−0.23, 0.31]	[−0.29, 0.28]	[−0.36, 0.21]	[−0.18, 0.51]	[−0.29, 0.40]	[−0.23, 0.50]	[−0.28, 0.46]
P. Pegboard.Non^h^	[−0.21, 0.34]	[−0.43, 0.09]	[−0.03, 0.51]	[−0.13, 0.43]	[−0.30, 0.40]	[−0.52, 0.14]	[−0.02, 0.65]	[−0.10, 0.59]
Block Design^i^	[−0.19, 0.42]	[−0.33, 0.26]	[−0.32, 0.32]	[−0.42, 0.21]	[−0.25, 0.53]	[−0.31, 0.45]	[−0.30, 0.53]	[−0.36, 0.48]
Aβ^j^	[−0.55, 0.13]	[−0.65, −0.04]	[−0.17, 0.56]	[−0.10, 0.60]	[−0.65, 0.24]	[−0.78, −0.07]	[−0.36, 0.64]	[−0.28, 0.69]
Tau^k^	[−0.72, −0.10]	[−0.51, 0.21]	[−0.40, 0.41]	[−0.36, 0.18]	[−0.72, 0.14]	[−0.63, 0.26]	[−0.60, 0.45]	[−0.55, 0.51]

^a^ IQ ≤ 45.

^b^ Preference scores indicate preference to fixate on the novel image of the test phase.

^c^ Dementia Questionnaire for People with Learning Disabilities sum of social score (Evenhuis 2018).

^d^ Dementia Questionnaire for People with Learning Disabilities sum of cognitive score (Evenhuis 2018).

^e^ Modified Cued Recall Test total score (Zimmerli and Devenny 1995).

^f^ Modified Cued Recall Test cued intrusions score (Zimmerli and Devenny 1995).

^g^ Purdue Pegboard dominant hand score (Vega 1969).

^h^ Purdue Pegboard nondominant hand score (Vega 1969).

^i^ Wechsler Intelligence Scale for Children Block Design subtest (Wechsler 2004) with Haxby downward extension (Haxby 1989).

^j^ PET amyloid‐beta.

^k^ PET tau.

#### 3.1.1 Gaze Samples

Average gaze samples for the full sample ranged from 7% to 88% (*M* = 56.8, *SD* = 21.3), indicating wide individual differences in ability to direct and sustain attention on target images (influencing learning). To ensure memory of images was captured, follow‐up analyses were conducted on the participants who had an average gaze sample ≥ 50% (*n* = 36). In this subset of participants, chronological age was again significantly correlated with average saccadic amplitude on novel images (*r* = −0.38, *p* = 0.023). Purdue Pegboard (nondominant hand only) was significantly associated with both visual preference scores (fixation count: *r* = 0.39, *p* = 0.023; fixation duration: *r* = 0.37, *p* = 0.035); that is, greater preference to fixate on the novel image in the test phase was related to better performance. The DLD‐SOS was significantly associated with preference to fixate on the novel image (fixation count: *r* = −0.59, *p* < 0.001; fixation duration: *r* = −0.56, *p* < 0.001). Finally, fixation duration during the habituation phases (*r* = −0.71, *p* = 0.022) was significantly associated with PET tau. All correlation coefficients for this subgroup (gaze sample ≥ 50%) are provided in Table S1.

#### 3.1.2 Subgroup With Lower IQ Scores

Table 2 provides means and standard deviations for the main variables for the subgroup with IQ ≤ 45 and those with IQ > 45. Table 3 shows the correlations for the subgroup of participants with IQs ≤ 45 (*n* = 33). Twenty (61%) participants in the subgroup with IQ scores ≤ 45 had an average gaze sample ≥ 50%. Chronological age was significantly associated with average saccadic amplitude for novel images (*r* = −0.48, *p* = 0.006). The mCRT total was significantly associated with average saccadic amplitude for novel images (*r* = 0.41, *p* = 0.040). Greater fixation duration during the habituation phases (*r* = −0.40, *p* = 0.047) and average saccadic amplitude for novel images (*r* = −0.39, *p* = 0.047) were significantly correlated with mCRT intrusions. The visual preference score for the novel images was significantly associated with the DLD‐SCS (fixation count: *r* = −0.41, *p* = 0.030). Additionally, visual preference score for the novel images was significantly associated with the DLD‐SOS score (fixation count: *r* = −0.64, *p* < 0.001; fixation duration: *r* = −0.60, *p* < 0.001). PET Aβ and tau were significantly associated with average saccadic amplitude for novel images (*r* = −0.55, *p* = 0.022 and *r* = −0.57, *p* = 0.021, respectively). PET tau was also significantly related to average fixation duration during the habituation phases (*r* = −0.59, *p* = 0.020). Remaining associations were not significant. A follow‐up independent *t* test indicated that participants with IQs > 45 fixated significantly longer during the habituation phases than those with IQs ≤ 45 (*t*[52] = −1.7, *p* = 0.047). The IQ groups did not significantly differ in saccadic amplitude or visual preference scores. Thus, when examining associations for the full sample versus those with gaze sample ≥ 50% subgroup and those with IQs ≤ 45, the relative magnitude and direction of associations between the eye‐movement metrics and ad pathology and symptomatology were similar.

### 3.2 Qualitative Analysis of Sample Gaze Plots

**FIGURE 2:**
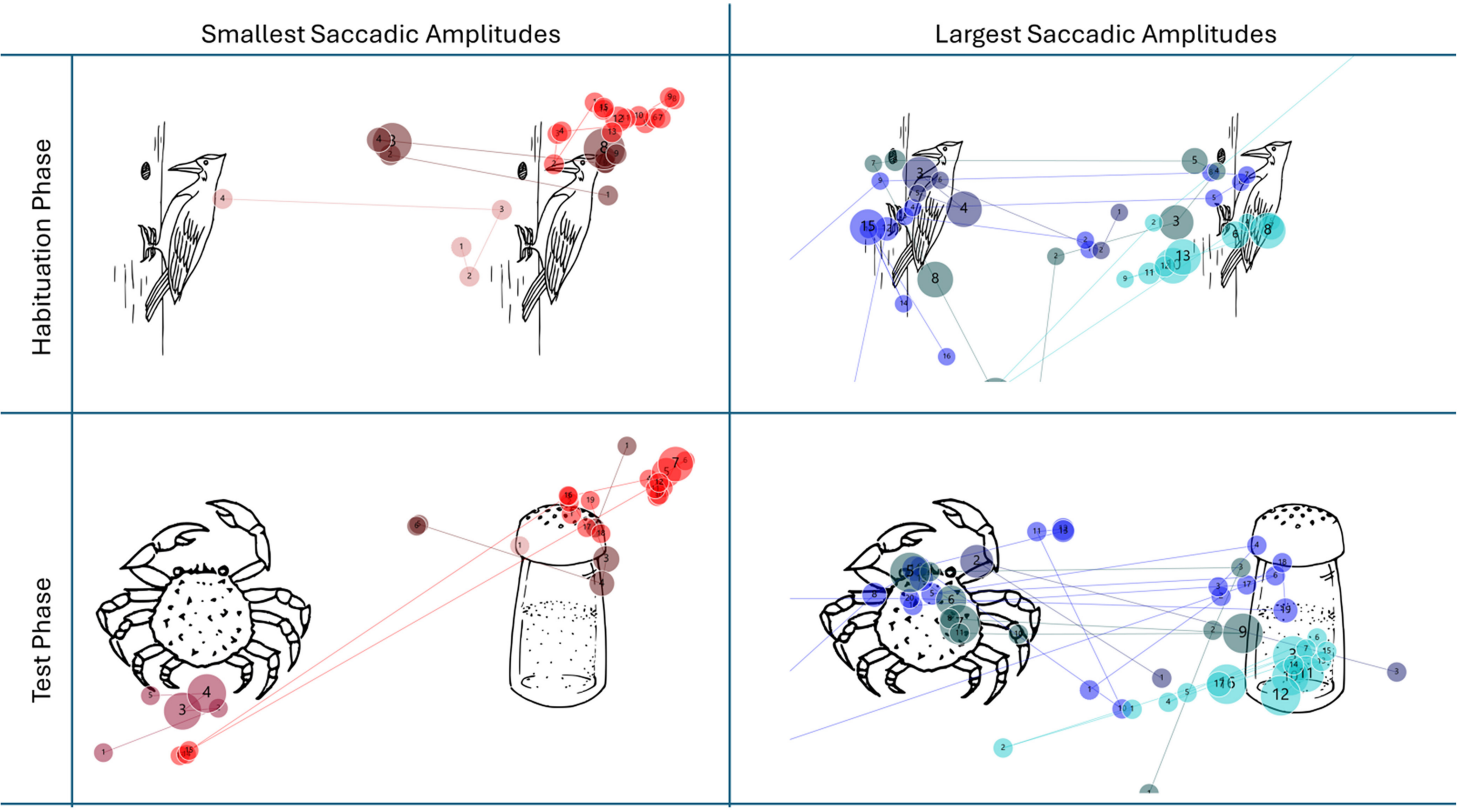
Gaze plots for Top 4 participants with the largest and Top 4 smallest saccadic amplitudes for both the habituation and the test phases.

**FIGURE 3:**
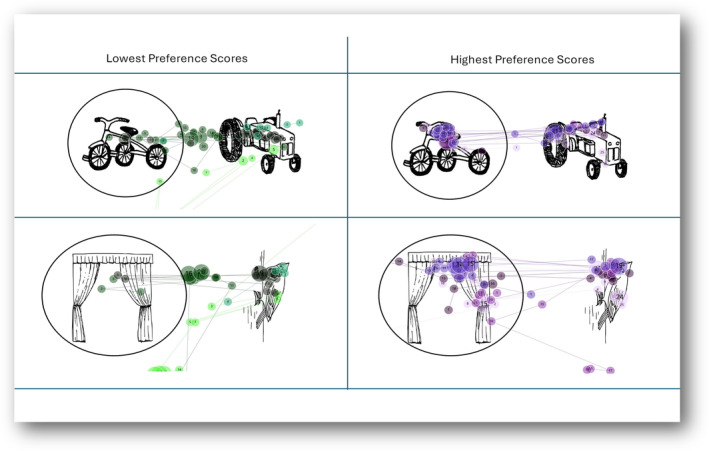
Gaze plots for Top 4 participants with highest and Top 4 lowest visual preference scores. Visual preference scores indicate the preference to fixate on the novel (as opposed to the familiar) image during the test phase. Novel images are circled.

To interpret saccade data, gaze plots for four participants with the largest average saccadic amplitudes and four participants with the smallest average saccadic amplitudes were reviewed (Figure 2). For the habituation phases, participants with the largest saccadic amplitudes shifted their gaze from one habituation image to the other, indicative of learning. In contrast, participants with the smallest saccadic amplitudes were often off‐target. During the test phases, participants with large saccadic amplitudes initially shifted their gaze to the novel image, followed by back and forth between the novel and familiar image. In contrast, participants with small saccadic amplitudes showed little image exploration and often had off‐target fixations. Figure 3 depicts gaze plots for four participants with the lowest visual preference scores and four participants with the highest visual preference scores. Those with low scores fixated in the middle or on the familiar image. In contrast, participants with high preference scores initially fixated on the novel image and then shifted their gaze to the familiar image.

## 4 Discussion

The present study was the first to examine whether an eye‐tracking VPC task was sensitive to early ad symptomatology and pathology in adults with DS with varying IQs. Current cognitive measures validated to detect ad‐related cognitive decline are generally limited to adults with DS with verbal communication and/or who have mild to moderate premorbid ID. Our findings suggest that eye‐tracking VPC tasks are feasible with adults with DS with IQs ranging from mild to severe/profound ID and sensitive to early ad pathology and symptomatology. In line with our hypothesis and consistent with research on late‐onset sporadic ad (e.g., Ionescu et al. 2023), differences in the number and duration of fixations and in saccadic amplitudes during both a learning and test phase differentiated adults with DS who were older and who had more ad pathology and/or symptomatology.

The pattern of eye movement differences associated with ad in DS, however, differed from that of the general adult population. In line with patterns associated with ad in the general adult population (Crutcher et al. 2009; Zola et al. 2013), shorter total fixation duration during the habituation phase and lower preference for the novel image based on fixation number and duration were associated with poorer memory (mCRT), more informant‐reported dementia symptoms (DLD‐SOS) and higher PET tau. Thus, for both populations, greater ad symptomatology and pathology are associated with poorer visual learning and memory. However, smaller saccadic amplitude for the novel image during the testing phase was associated with older chronological age and higher PET Aβ accumulation and more memory impairments (mCRT) in DS. This pattern remained when limiting the sample to those with ≥ 50% gaze samples and thus for whom attention was largely on target images. Moreover, this pattern was largely replicated when considering only adults with DS with IQs ≤ 45. Of note, in this group with lower IQ scores, new associations emerged between having smaller saccadic amplitude for the novel images and more tau PET burden as well as between greater visual preference scores (fixation count) and fewer dementia symptoms (DLD‐SCS). It is also important to note that there were only differences in the habituation phase (not test phase) when comparing eye‐tracking metrics between adults with DS with IQs ≤ 45 versus > 45. This suggests that eye‐tracking metrics can pick up on differences in learning across IQ levels.

The ‘DS‐specific pattern’ of altered eye movements in relation to ad may reflect the lower baseline developmental level of adults with DS. Neurotypical younger children (e.g., < 10 years) spend more time in the ambient attentional processing phase with larger saccadic amplitudes due to a global scanning strategy (Helo et al. 2014). As a neurotypical child's cognitive development matures, saccadic amplitudes become smaller, with a quicker shift to longer fixation durations to capture stimulus details, employing the more advanced focal mode of attentional processing. In the current study, adults with DS had an average mental age equivalency of 5.0 years (*SD* = 1.4). Thus, attentional processing strategies of adults with DS may more closely mirror those of neurotypical children than adults. As a result, larger saccadic amplitudes may reflect intact learning and memory functioning for adults with DS. Indeed, a review of gaze plots suggested that adults with DS with large saccadic amplitudes and high visual preference scores evidenced patterns conducive to learning (gaze shifting between habituation images) and were initially drawn to novel images. In contrast, adults with DS with small saccadic amplitudes and low visual preference scores tended to miss the habituation image altogether and evidenced little image exploration during testing phases. It should be noted that in prior studies (Rose et al. 2013; van Herwegen 2015) shorter saccadic amplitude and longer fixations were reported for individuals with ID relative to neurotypical individuals and interpreted as being reflective of lower learning associated with ID. In our sample, large saccadic amplitude was negatively associated with signs of ad in those with IQs ≤ and > 45, with no mean difference in saccades between these groups, suggesting that this profile is a sign of ad and not severity of ID.

### 4.1 Strengths, Limitations and Future Directions

The present study's use of direct‐ and informant‐reported measures to examine associations between eye‐movement metrics and ad pathology and symptomatology in adults with DS strongly contributes to the field; however, there are limitations. The small sample size hinders our ability to detect associations with adequate statistical power, and therefore, findings should be interpreted with caution. However, the present study provides an important first step in the examination of eye‐tracking metrics and ad‐related cognitive functioning in DS and underscores the need for larger sample sizes in future studies. Additionally, we compared the VPC task to performance on cognitive measures deemed to have high sensitivity to early ad and low floor effects in DS samples; however, it should be noted that these tools do have limitations with adults with moderate to severe ID (Hartley et al. 2022; Krinsky‐McHale et al. 2022). On the VPC task, images were presented for 7 s, which is longer than what is typical for the general adult population (e.g., Zola et al. 2013) but may be too short for adults with DS to fully encode visual information. Additionally, accuracy and precision scores were low during the 9‐point calibration. However, the AOIs for images were large; thus, the accuracy and precision scores were acceptable. Future research should test simpler calibration strategies. It is also important to acknowledge that the use of the eye tracking device and accompanying software used in the present study requires training and financial resources that may prohibit their use in clinical settings. Future research should explore more affordable eye tracking strategies such as webcam eye tracking or head worn devices. Finally, whereas participants in the present study did not have a diagnosis of ad, future studies should include adults with DS with suspected or diagnosed ad to compare eye movement patterns and to determine sensitivity of the VPC task for adults with DS with ad.

## 5 Conclusion

Eye‐tracking technology is a potentially useful, noninvasive and less cumbersome tool for assessing cognitive functioning in adults with DS with varying IQ levels. Establishing valid tools for detecting ad‐related cognitive changes in adults with DS with a broad range of ID levels has important clinical implications. Such tools would advance more timely screening and diagnosis of ad, increase access to ad treatments and clinical trials and could be used to inform care management plans when early memory and learning problems arise. Findings from the present study revealed a robust pattern of associations between eye movements and ad cognitive impairments and biomarkers of early ad pathology. A lack of a preference for novel images and smaller amplitudes was associated with older age, greater ad pathology and greater cognitive impairments. Future larger sized and longitudinal studies are needed to replicate findings. Overall, findings suggest that eye‐tracking VPC tasks are useful for tracking early ad in adults with DS who have widely varying IQs.

## Conflicts of Interest

The authors declare no conflicts of interest.

## Supporting information


**Table S1.** Pearson Correlations for Participants with a Gaze Sample of ≥ 50%.

## Data Availability

The data that support the findings of this study are openly available in ABC‐DS at https://abc‐ds.org/researchers/.
