# Supplementary material for: Eye Tracking as a Tool for Detecting Alzheimer's Disease in People With Down Syndrome
Source: J Intellect Disabil Res. 2025 Feb 10;69(5):370–82. doi: 10.1111/jir.13214 (PMC11966356; doi:10.1111/jir.13214)
Supplement: Supplementary file 1 — Table S1. Pearson Correlations for Participants with a Gaze Sample of ≥ 50%. [file JIR-69-370-s001.docx]

|  | **Fixation**  **Duration**  **(Habituation Phase)** | **Saccadic**  **Amplitude_**  **Novel (test)** | **Preference^1^_ Fixation**  **Count (test)** | **Preference_**  **Fixation**  **Duration (test)** |
| --- | --- | --- | --- | --- |
| **Age** | .029 | **-.382^*^** | .073 | .071 |
| **Sex** | .060 | .070 | -.126 | -.104 |
| **DLD-SOS^2^** | -.044 | .135 | **-.585^***^** | **-.557^***^** |
| **DLD-SCS^3^** | -.145 | -.034 | -.282 | -.230 |
| **mCRT total^4^** | -.085 | .210 | -.019 | -.042 |
| **mCRT intrusions^5^** | -.109 | -.099 | .128 | .138 |
| **P.Pegboard.Dom^6^** | .294 | .032 | .123 | .091 |
| **P. Pegboard.Non^7^** | .041 | -.236 | **.394^*^** | **.369^*^** |
| **Block Design^8^** | .223 | -.185 | .140 | .106 |
| **Aβ^9^** | -.282 | -.348 | .477 | .440 |
| **Tau^10^** | **-.706^*^** | -.158 | .190 | .157 |

Supplementary Table 1. Pearson Correlations for Participants with a Gaze Sample of ≥ 50%

Note: ^1^ Preference scores indicate preference to fixate on the novel image of the test phase; ^2^Dementia Questionnaire for People with Learning Disabilities sum of social score (Evenhuis et al., 2018); ^3^Dementia Questionnaire for People with Learning Disabilities sum of cognitive score (Evenhuis et al., 2018); ^4^modified Cued Recall Test total score (Zimmerli & Devenney, 1995); ^5^modified Cued Recall Test cued intrusions score (Zimmerli & Devenney, 1995); ^6^Purdue Pegboard dominant hand score (Vega, 1969); ^7^Purdue Pegboard nondominant hand score (Vega, 1969); ^8^Wechsler Intelligence Scale for Children Block Design subtest (Wechsler, 2004) with Haxby downward extension (Haxby, 1989); ^9^PET amyloid-beta; ^10^PET tau.
